# Dissecting causal relationships between immune cells, plasma metabolites, and COPD: a mediating Mendelian randomization study

**DOI:** 10.3389/fimmu.2024.1406234

**Published:** 2024-05-28

**Authors:** Zhenghua Cao, Tong Wu, Yakun Fang, Feng Sun, Huan Ding, Lingling Zhao, Li Shi

**Affiliations:** ^1^ Graduate School, Changchun University of Traditional Chinese Medicine, Changchun, Jilin, China; ^2^ Respiratory Disease Department, Affiliated Hospital of Changchun University of Traditional Chinese Medicine, Changchun, Jilin, China

**Keywords:** Mendelian randomization, immune cells, plasma metabolites, COPD, mediation analysis

## Abstract

**Objective:**

This study employed Mendelian Randomization (MR) to investigate the causal relationships among immune cells, COPD, and potential metabolic mediators.

**Methods:**

Utilizing summary data from genome-wide association studies, we analyzed 731 immune cell phenotypes, 1,400 plasma metabolites, and COPD. Bidirectional MR analysis was conducted to explore the causal links between immune cells and COPD, complemented by two-step mediation analysis and multivariable MR to identify potential mediating metabolites.

**Results:**

Causal relationships were identified between 41 immune cell phenotypes and COPD, with 6 exhibiting reverse causality. Additionally, 21 metabolites were causally related to COPD. Through two-step MR and multivariable MR analyses, 8 cell phenotypes were found to have causal relationships with COPD mediated by 8 plasma metabolites (including one unidentified), with 1-methylnicotinamide levels showing the highest mediation proportion at 26.4%.

**Conclusion:**

We have identified causal relationships between 8 immune cell phenotypes and COPD, mediated by 8 metabolites. These findings contribute to the screening of individuals at high risk for COPD and offer insights into early prevention and the precocious diagnosis of Pre-COPD.

## 1 Introduction

Chronic Obstructive Pulmonary Disease (COPD) is a heterogeneous disorder primarily characterized by airway pathologies (bronchitis, bronchiolitis) and/or alveolar abnormalities (emphysema), leading to chronic respiratory symptoms (dyspnea, cough, sputum production) and progressively worsening airflow limitation (1). Globally, COPD accounts for more than half of all chronic respiratory disease cases (2), gradually becoming the third leading cause of death worldwide (3). With the increasing prevalence of an aging population, both the incidence and mortality rates of COPD are on the rise annually (4), imposing a significant economic burden on society (5).

Immune cells possess multifaceted functions in maintaining homeostasis and facilitating repair after injury (6). The lungs may serve as a battleground for the interaction between various microbes and the host’s innate and adaptive immune defenses (7). Consequently, the immune system could be a pivotal driving force in the pathogenesis of COPD, with immune responses being significantly associated with acute exacerbations of COPD (8). However, the detailed physiological mechanisms remain insufficiently explored (9). Most existing evidence, primarily from observational studies, indicates that compared to healthy controls, individuals with COPD have an increased presence of immune cells in lung tissue (10, 11) and an upregulated immune cell response (12). Cells such as CD68+ myeloid antigen-presenting cells, CD4+ T cells, and CD8 T cells are found to proliferate in the lungs of patients with COPD, potentially leading to persistent inflammation (13, 14). Certain immune cells exhibit a negative correlation with the frequency of COPD exacerbations (15), such as CD4 T cells and resting natural killer cells (16). Consequently, the causal relationship and underlying mechanisms between immune cells and COPD remain unclear. Metabolites, as intermediates of metabolic reactions, can influence disease progression (17) and serve as targets for therapeutic intervention (18). They have the potential to improve the diagnosis and treatment of COPD (19) and may play a synergistic role in its pathogenesis (20), possibly mediating important immunoregulatory functions (21). Compared to healthy controls, COPD patients exhibit reduced levels of the metabolites 1-methylnicotinamide creatinine, and lactate (17). Glutamylphenylalanine may serve as a biomarker for acute exacerbations of COPD (22), while sphingolipids are associated with pulmonary function (23). Therefore, we hypothesize a causal relationship between immune cells, metabolites, and COPD. Elucidating these associations and understanding the true causal relationships among immune cells, metabolites, and COPD could aid in the early identification, prevention, and management of COPD.

Mendelian Randomization (MR) represents a potential method for causal inference, designed to estimate the causal effects of exposure factors on outcomes, with the capability to control for potential confounding factors and circumvent reverse causation biases (24). Utilizing the methodology of MR, we are poised to conduct a bidirectional MR study concerning immune cells and COPD, concurrently undertaking two mediating analyses to dissect the causal relationships among immune cells, metabolites, and COPD.

## 2 Methods

### 2.1 Study design

**Figure 1:**
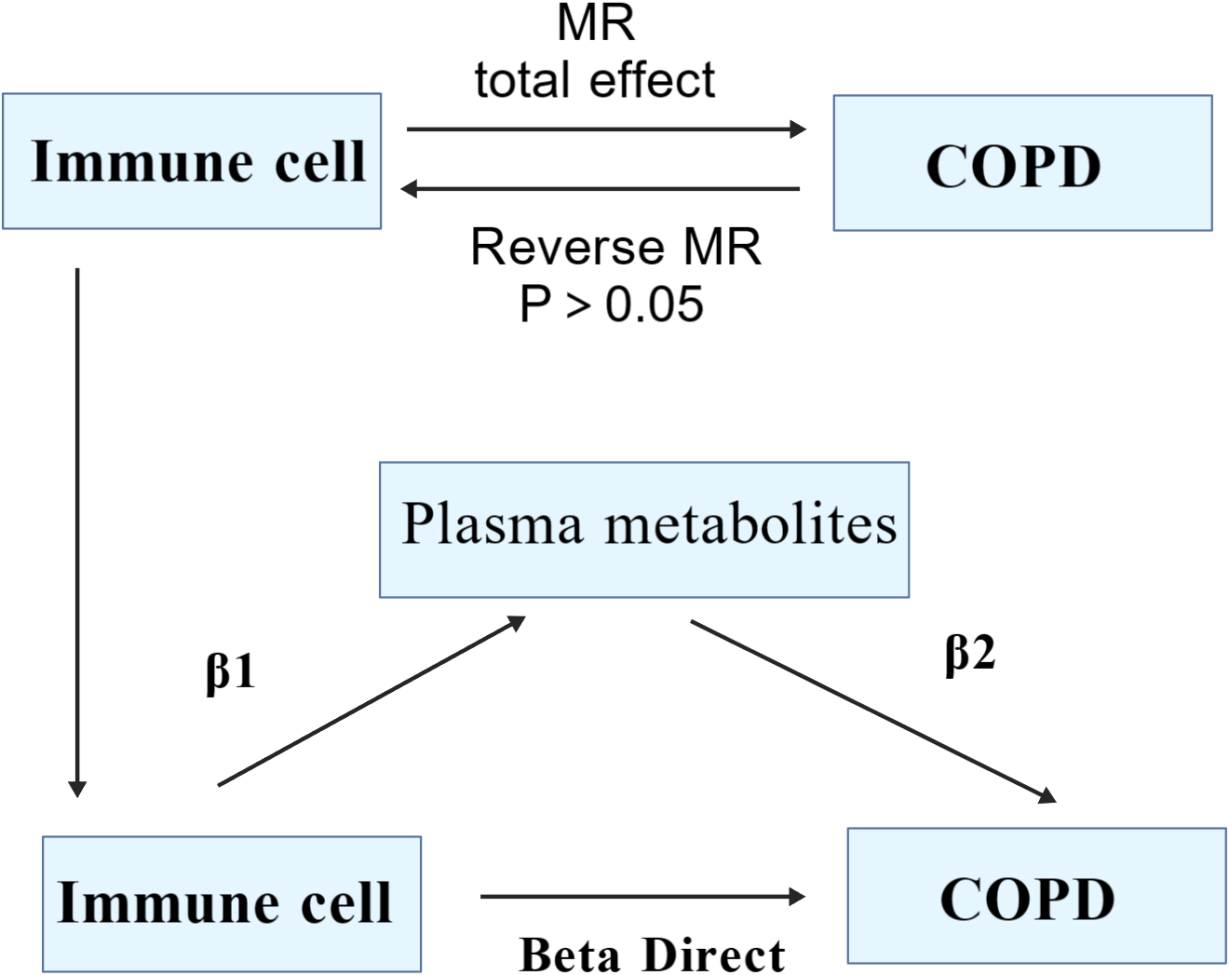
Research ideas.

Grounded in two-sample Mendelian Randomization, our study initially assessed the causal relationship between 731 immune cell phenotypes across 7 panels (B cell, cDC, TBNK, Treg, Myeloid cell, Maturation stages of T cell and Monocyte) and COPD. Proceeding with COPD as the exposure factor and employing the Inverse Variance Weighted (IVW) method to select immune cells as the outcome factor, we conducted reverse Mendelian Randomization to ascertain the presence of a reverse causal relationship. Utilizing both the two-step MR (TSMR) and multivariable MR approaches, with 1400 plasma metabolites serving as mediating factors, we aimed to elucidate the significant mediatory role that plasma metabolites may play in the causal pathway between immune cells and COPD (**Figure 1**).

### 2.2 Data sources

The genetic information pertinent to COPD was sourced from the GWAS database (https://gwas.mrcieu.ac.uk/), with the selected dataset bearing the identifier ebi-a-GCST90018807, encompassing 468,475 samples and 24,180,654 SNPs, all of which pertain to the European population. The genetic data related to 731 immune cell phenotypes were derived from a 2020 study (25), all pertaining to the European demographic, with the catalog identifiers ranging from ebi-a-GCST90001391 to ebi-a-GCST90002121. The GWAS data for 1,400 plasma metabolites, hailing from a 2023 study (18), are accessible from the GWAS database, with identifiers spanning from GCST90199621 to GCST90201020, all associated with the European population.

### 2.3 Instrumental variable selection

The selection of instrumental variables necessitates adherence to several assumptions (26), to fulfill their relevance (27), we conducted an association analysis on 731 immune cell phenotypes and 1,400 plasma metabolites, uniformly applying a threshold of P<1×10^−5^ (28, 29). Subsequently, SNPs exhibiting linkage disequilibrium were filtered out using criteria of R^2<0.001 and Kb=10,000 (30), followed by the calculation of the F-statistic for the selected SNPs to eliminate weak instrumental variables. An F-statistic greater than 10 is considered indicative of the absence of weak instrumental variables (31, 32).

### 2.4 Statistical analysis

We employed five methodologies to assess causality: Inverse Variance Weighted (IVW), MR-Egger, Weighted Median, Simple Mode, and Weighted Mode methods, with IVW serving as the primary approach (33, 34). P<0.05 was indicative of a causal relationship (35), while the other four methods served as supplementary analyses (36). To evaluate the robustness of our results, we conducted sensitivity analysis using the “leave-one-out” approach, further examining pleiotropy and heterogeneity, P>0.05 suggesting the absence of both (37, 38). Utilizing the TSMR approach, we first calculated the total effect from immune cells to COPD, the effect of immune cells on metabolites (β1), and the effect of metabolites on COPD (β2), followed by the calculation of the mediating effect (β1*β2), with the direct effect being the total effect minus the mediating effect (39). All analyses were conducted using the R language (version 4.3.2), with the TwoSampleMR package at version 0.6.0.

## 3 Results

### 3.1 Genetic causality between immune cells and COPD

**Figure 2:**
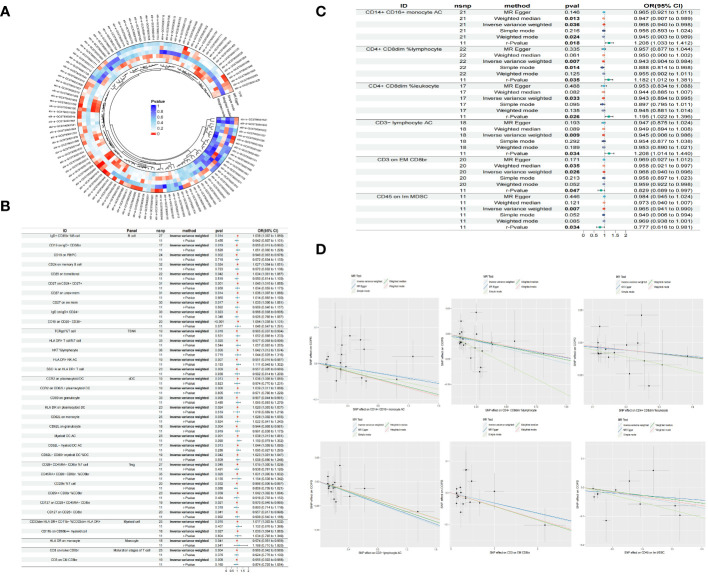
Circle plot of five Mendelian randomized methods (P<0.05) **(A)** Forest plot of causality between 35 immune cells and COPD (r-Pvalue is the result of reverse MR) **(B)** Forest plot of causality between six immune cells and COPD **(C)** Scatter plot of six immune cells reducing COPD risk **(D)**.

Through the selection of quantitative tools, we conducted an associative analysis, eliminating linkage disequilibrium and weak instrumental variables, thereby identifying 13,318 SNPs associated with immune cells, with the smallest F-value being 19.53. Preliminary investigations via the Inverse Variance Weighted (IVW) method revealed 41 immune cell phenotypes correlated with COPD, including but not limited to IgD+ CD38br %B cell, CD19 on IgD- CD38br, CD19 on PB/PC, and CD24 on memory B cell within the B cell category; TCRgd %T cell, HLA DR+ T cell%T cell, NKT %lymphocyte, and HLA DR+ NK AC within the TBNK category; CCR2 on plasmacytoid DC, CCR2 on CD62L+ plasmacytoid DC, CD80 on granulocyte, and CD62L on monocyte within the cDC category; and CD28+ CD45RA- CD8br %T cell, CD45RA+ CD28- CD8br %CD8br, CD25hi %T cell, and CD25++ CD8br %CD8br within the Treg category. In our study, we conducted a reverse Mendelian randomization analysis with COPD as the exposure factor and 41 immune cell phenotypes as the outcome factors. Our findings revealed that COPD does not exhibit a reverse causal relationship with 35 of the immune cell phenotypes (r-Pvalue > 0.05). However, a reverse causality was observed in six immune cell phenotypes (r-Pvalue < 0.05), specifically CD14+ CD16+ monocyte AC, CD4+ CD8dim %lymphocyte, CD4+ CD8dim %leukocyte, CD3- lymphocyte AC, CD3 on EM CD8br, and CD45 on Im MDSC. Furthermore, 21 immune cell phenotypes demonstrated a negative correlation with COPD, while 20 showed a positive correlation. Concurrently, tests for pleiotropy and heterogeneity yielded results (P> 0.05), with the direction of OR values being consistent, and leave-one-out sensitivity analysis confirmed the robustness of the MR findings (**Table 1**, **Figure 2**).

**Table 1:** MR analysis of immune cells and COPD.

Exposure	Method	Nsnp	Beta	Se	P-val	Pleiotropy	Heterogeneity
IgD+ CD38br %B cell	IVW	27	0.037	0.015	0.014	0.780	0.789
Myeloid DC AC	IVW	23	0.037	0.012	0.001	0.605	0.733
CD62L- myeloid DC AC	IVW	17	0.043	0.017	0.013	0.731	0.363
CD62L- CD86+ myeloid DC %DC	IVW	19	0.023	0.011	0.042	0.156	0.762
CD25hi %T cell	IVW	20	-0.034	0.016	0.032	0.901	0.468
CD33dim HLA DR+ CD11b- %CD33dim HLA DR+	IVW	25	0.017	0.007	0.016	0.487	0.840
CD14+ CD16+ monocyte AC	IVW	21	-0.032	0.015	0.036	0.843	0.487
CD4+ CD8dim %lymphocyte	IVW	22	-0.058	0.022	0.007	0.716	0.101
CD4+ CD8dim %leukocyte	IVW	17	-0.059	0.027	0.033	0.867	0.053
TCRgd %T cell	IVW	19	-0.036	0.015	0.018	0.363	0.523
HLA DR+ T cell%T cell	IVW	33	-0.023	0.010	0.020	0.348	0.622
NKT %lymphocyte	IVW	36	0.041	0.015	0.006	0.225	0.180
CD3- lymphocyte AC	IVW	18	-0.057	0.022	0.009	0.954	0.947
HLA DR+ NK AC	IVW	19	-0.050	0.019	0.007	0.648	0.720
CD25++ CD8br %CD8br	IVW	23	0.042	0.020	0.039	0.792	0.523
CD28+ CD45RA- CD8br %T cell	IVW	27	0.014	0.007	0.045	0.488	0.355
CD45RA+ CD28- CD8br %CD8br	IVW	35	0.001	0.000	0.020	0.937	0.721
CD19 on CD20- CD38-	IVW	20	0.080	0.022	0.000	0.587	0.116
CD19 on IgD- CD38br	IVW	17	-0.046	0.020	0.019	0.778	0.452
CD19 on PB/PC	IVW	24	-0.062	0.020	0.002	0.101	0.258
CD24 on memory B cell	IVW	32	0.027	0.012	0.024	0.270	0.263
CD25 on transitional	IVW	22	0.033	0.016	0.042	0.579	0.927
CD27 on CD24+ CD27+	IVW	31	0.039	0.012	0.001	0.975	0.196
CD27 on unsw mem	IVW	31	0.036	0.015	0.014	0.521	0.340
CD27 on sw mem	IVW	30	0.032	0.014	0.017	0.265	0.306
IgD on IgD+ CD24-	IVW	30	-0.034	0.015	0.023	0.810	0.459
CD62L on monocyte	IVW	25	0.028	0.013	0.036	0.238	0.883
CD62L on granulocyte	IVW	18	-0.058	0.020	0.004	0.230	0.476
CD3 on naive CD8br	IVW	25	-0.035	0.012	0.004	0.948	0.972
CD3 on EM CD8br	IVW	20	-0.033	0.015	0.026	0.953	0.507
CD3 on CM CD8br	IVW	19	-0.046	0.017	0.008	0.549	0.956
CD127 on CD28+ CD45RA- CD8br	IVW	19	-0.030	0.013	0.021	0.237	0.618
CD127 on CD28- CD8br	IVW	20	-0.044	0.022	0.041	0.246	0.618
HLA DR on monocyte	IVW	18	-0.026	0.013	0.041	0.558	0.077
CCR2 on plasmacytoid DC	IVW	19	0.035	0.014	0.013	0.765	0.423
CCR2 on CD62L+ plasmacytoid DC	IVW	19	0.039	0.014	0.006	0.447	0.550
CD80 on granulocyte	IVW	33	-0.033	0.012	0.008	0.110	0.798
CD45 on Im MDSC	IVW	11	-0.036	0.013	0.007	0.271	0.391
SSC-A on HLA DR+ T cell	IVW	23	-0.044	0.017	0.009	0.673	0.511
CD11b on CD66b++ myeloid cell	IVW	18	0.032	0.015	0.027	0.209	0.540
HLA DR on plasmacytoid DC	IVW	23	0.019	0.009	0.024	0.146	0.339

### 3.2 Genetic causality between metabolites and COPD

**Figure 3:**
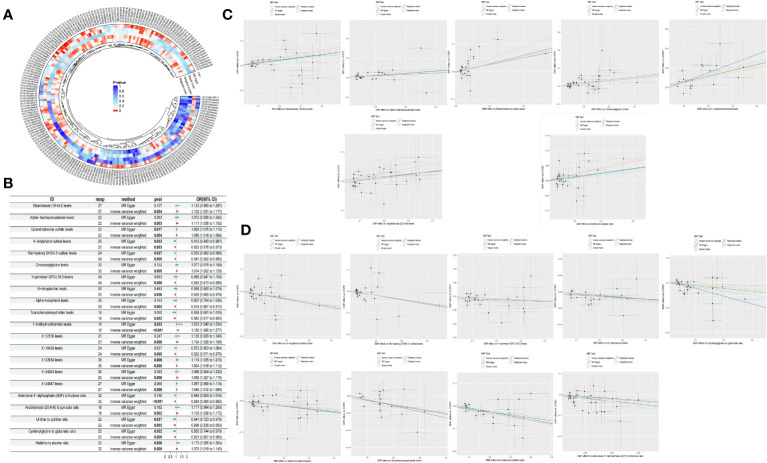
Circle plot of five Mendelian randomization methods (p <0.05) **(A)** Forest plot of causality of 21 metabolites and COPD **(B)** Scatter plot of 7 metabolites with increased COPD risk **(C)** Scatter plot of the 9 metabolites reducing the risk of COPD **(D)**.

Through the selection of instrumental variables, we conducted an association analysis, eliminating linkage disequilibrium and weak instrumental variables, thereby identifying 29,302 SNPs associated with plasma metabolites, with the smallest F-statistic being 19.50. The IVW method preliminarily identified 21 plasma metabolites causally related to COPD, comprising 16 known metabolites and 5 unknown. Among the known metabolites, 7 were potentially associated with an increased risk of COPD, namely Stearidonate (18:4n3), Alpha-hydroxyisovalerate, Epiandrosterone sulfate, Cinnamoylglycine, 1-methylnicotinamide, the Arachidonate (20:4n6) to pyruvate ratio, and the Histidine to alanine ratio. Conversely, 9 metabolites were potentially inversely correlated with COPD risk, including 4-vinylphenol sulfate, 16a-hydroxy DHEA 3-sulfate, 1-palmitoyl-GPG (16:0), N-oleoylserine, Alpha-tocopherol, Taurochenodeoxycholate, the Adenosine 5’-diphosphate (ADP) to fructose ratio, the Uridine to cytidine ratio, and the Cysteinylglycine to glutamate ratio (**Table 2**, **Figure 3**).

**Table 2:** MR analysis of metabolites and COPD.

Exposure	Method	Nsnp	Beta	Se	P-val	Pleiotropy	Heterogeneity
Stearidonate (18:4n3) levels	IVW	27	0.097	0.034	0.004	0.751	0.304
Alpha-hydroxyisovalerate levels	IVW	22	0.106	0.036	0.003	0.591	0.055
Epiandrosterone sulfate levels	IVW	23	0.055	0.019	0.004	0.650	0.393
4-vinylphenol sulfate levels	IVW	25	-0.080	0.027	0.003	0.692	0.224
16a-hydroxy DHEA 3-sulfate levels	IVW	24	-0.061	0.021	0.005	0.667	0.407
Cinnamoylglycine levels	IVW	33	0.071	0.025	0.005	0.946	0.984
1-palmitoyl-GPG (16:0) levels	IVW	24	-0.078	0.029	0.008	0.462	0.583
N-oleoylserine levels	IVW	20	-0.075	0.027	0.006	0.568	0.984
Alpha-tocopherol levels	IVW	28	-0.086	0.029	0.003	0.845	0.656
Taurochenodeoxycholate levels	IVW	18	-0.124	0.040	0.002	0.563	0.521
1-methylnicotinamide levels	IVW	18	0.168	0.039	0.000	0.486	0.594
X-12100 levels	IVW	21	0.099	0.038	0.008	0.869	0.293
X-19438 levels	IVW	24	-0.081	0.029	0.005	0.335	0.861
X-23654 levels	IVW	39	0.062	0.023	0.006	0.131	0.944
X-24243 levels	IVW	20	0.094	0.034	0.006	0.645	0.834
X-24947 levels	IVW	27	0.047	0.018	0.008	0.714	0.446
Adenosine 5’-diphosphate (ADP) to fructose ratio	IVW	32	-0.079	0.020	0.000	0.409	0.622
Arachidonate (20:4n6) to pyruvate ratio	IVW	16	0.098	0.031	0.002	0.848	0.868
Uridine to cytidine ratio	IVW	22	-0.110	0.035	0.002	0.374	0.477
Cysteinylglycine to glutamate ratio	IVW	25	-0.082	0.031	0.009	0.228	0.569
Histidine to alanine ratio	IVW	32	0.076	0.029	0.009	0.080	0.280

### 3.3 Mediated Mendelian randomization analysis

**Figure 4:**
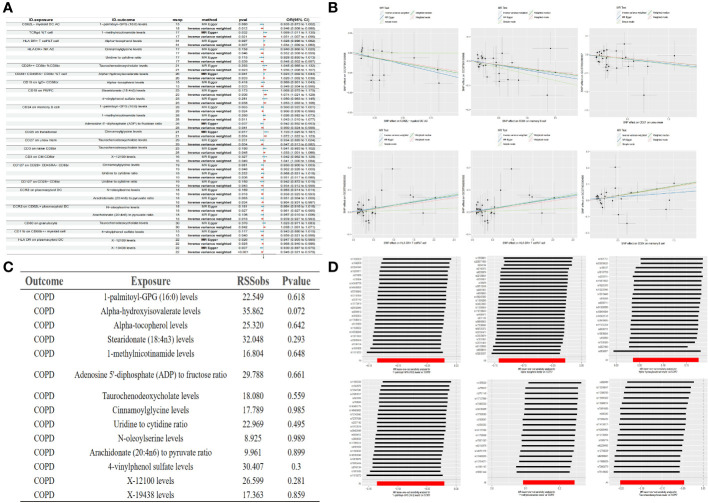
Forest plot of immune cells and metabolites **(A)** Partial scatter plot of immune cells and metabolites **(B)** Test of MR-PRESSO of metabolites to COPD **(C)** Partial leave-one-out method sensitivity analysis of metabolites to COPD **(D)**.

Building upon the previously identified immune cells and plasma metabolites, we employed the TSMR approach to further compute mediation through Mendelian randomization. Utilizing the 35 selected immune cell phenotypes as exposure factors and the 21 plasma metabolites as outcome measures, we conducted a MR analysis from immune cell phenotypes to plasma metabolites. This analysis revealed causal relationships between 20 immune cell phenotypes and 14 plasma metabolites, yielding the effect size β1 from immune cell phenotypes to metabolites. Our research has uncovered that there is a negative correlation between CD62L- myeloid DC AC and 1-palmitoyl-GPG (16:0), a positive correlation between TCRgd %T cell and 1-methylnicotinamide, and a positive correlation between HLA DR+ T cell%T cell and Alpha-tocopherol levels, among other findings. Further analysis has revealed that a single immune cell phenotype can have causal relationships with multiple metabolites. For instance, HLA DR+ NK AC not only exhibits a negative correlation with Cinnamoylglycine but also with the Uridine to cytidine ratio. Similarly, CD19 on PB/PC shows a positive correlation with Stearidonate (18:4n3) as well as with 4-vinylphenol sulfate. Moreover, CD24 on memory B cell not only negatively correlates with 1-palmitoyl-GPG (16:0) and the Adenosine 5’-diphosphate (ADP) to fructose ratio but also positively correlates with 1-methylnicotinamide, among others. When considering the 14 plasma metabolites as exposure factors and COPD as the outcome, an MR analysis was conducted along with an MR-PRESSO test (p > 0.05), indicating no pleiotropy and unbiased SNPs. This led to the determination of the effect size β2 from metabolites to COPD, and subsequently, the overall effect from immune cells to COPD was calculated (**Figure 4**, **Table 3**).

**Table 3:** MR analysis of immune cells and metabolites.

Exposure	Outcome	Method	Nsnp	Beta	Se	P-val	Pleiotropy	Heterogeneity
CD62L- myeloid DC AC	1-palmitoyl-GPG (16:0) levels	IVW	15	-0.055	0.022	0.012	0.676	0.764
TCRgd %T cell	1-methylnicotinamide levels	IVW	17	0.049	0.022	0.021	0.360	0.855
HLA DR+ T cell%T cell	Alpha-tocopherol levels	IVW	31	0.034	0.013	0.007	0.575	0.709
HLA DR+ NK AC	Cinnamoylglycine levels	IVW	17	-0.049	0.024	0.046	0.700	0.764
HLA DR+ NK AC	Uridine to cytidine ratio	IVW	17	-0.053	0.026	0.039	0.553	0.371
CD25++ CD8br %CD8br	Taurochenodeoxycholate levels	IVW	21	0.055	0.024	0.023	0.753	0.957
CD28+ CD45RA- CD8br %T cell	Alpha-hydroxyisovalerate levels	IVW	26	0.019	0.009	0.033	0.552	0.830
CD19 on IgD- CD38br	Alpha-tocopherol levels	IVW	15	-0.052	0.025	0.033	0.471	0.782
CD19 on PB/PC	Stearidonate (18:4n3) levels	IVW	23	0.071	0.026	0.006	0.888	0.244
CD19 on PB/PC	4-vinylphenol sulfate levels	IVW	23	0.052	0.025	0.038	0.938	0.531
CD24 on memory B cell	1-palmitoyl-GPG (16:0) levels	IVW	28	-0.034	0.015	0.024	0.673	0.723
CD24 on memory B cell	1-methylnicotinamide levels	IVW	28	0.042	0.016	0.011	0.323	0.175
CD24 on memory B cell	Adenosine 5’-diphosphate (ADP) to fructose ratio	IVW	28	-0.040	0.020	0.041	0.306	0.589
CD25 on transitional	Cinnamoylglycine levels	IVW	21	0.069	0.024	0.004	0.329	0.338
CD27 on unsw mem	Taurochenodeoxycholate levels	IVW	29	-0.054	0.019	0.004	0.828	0.486
CD3 on naive CD8br	Taurochenodeoxycholate levels	IVW	25	0.033	0.016	0.045	0.753	0.770
CD3 on CM CD8br	X-12100 levels	IVW	16	0.041	0.021	0.049	0.987	0.750
CD127 on CD28+ CD45RA- CD8br	Cinnamoylglycine levels	IVW	19	-0.039	0.020	0.048	0.525	0.155
CD127 on CD28+ CD45RA- CD8br	Uridine to cytidine ratio	IVW	19	-0.051	0.018	0.006	0.315	0.310
CD127 on CD28- CD8br	Uridine to cytidine ratio	IVW	19	-0.047	0.023	0.043	0.697	0.379
CCR2 on plasmacytoid DC	N-oleoylserine levels	IVW	18	-0.043	0.018	0.018	0.802	0.876
CCR2 on plasmacytoid DC	Arachidonate (20:4n6) to pyruvate ratio	IVW	18	-0.037	0.017	0.034	0.480	0.668
CCR2 on CD62L+ plasmacytoid DC	N-oleoylserine levels	IVW	18	-0.040	0.018	0.027	0.838	0.775
CCR2 on CD62L+ plasmacytoid DC	Arachidonate (20:4n6) to pyruvate ratio	IVW	18	-0.041	0.018	0.018	0.894	0.881
CD80 on granulocyte	Taurochenodeoxycholate levels	IVW	30	0.035	0.017	0.042	0.655	0.478
CD11b on CD66b++ myeloid cell	4-vinylphenol sulfate levels	IVW	15	-0.042	0.020	0.040	0.558	0.594
HLA DR on plasmacytoid DC	X-12100 levels	IVW	22	-0.033	0.015	0.025	0.228	0.222
HLA DR on plasmacytoid DC	X-19438 levels	IVW	22	-0.052	0.015	0.001	0.282	0.696

### 3.4 Mediation analysis

**Figure 5:**
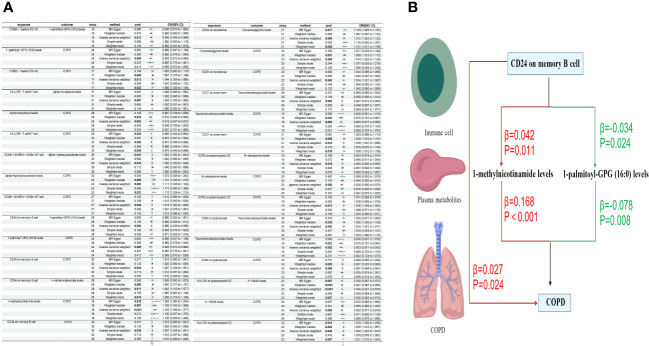
Forest plots of immune cells, plasma metabolites, and COPD **(A)** Plasma metabolites mediate the causal relationship between immune cells and COPD (red is a risk factor, green is a protective factor) **(B)**.

In our final analysis, we conducted a mediation analysis to elucidate the causal relationship between immune cell phenotypes and COPD, mediated by plasma metabolites. We discovered that 8 plasma metabolites mediated the relationship between 8 immune cell phenotypes and COPD (P < 0.05), among which 7 are known plasma metabolites and one remains unidentified. Notably, the CD24 on memory B cells was mediated by two distinct plasma metabolites. The mediation proportion of 1-methylnicotinamide was found to be the highest at 26.4% (P=0.013), followed by the unidentified metabolite X-19438 with a mediation proportion of 21.8% (P=0.004), Taurochenodeoxycholate at 18.8% (P=0.008), and Alpha-hydroxyisovalerate at 14.2% (P=0.036), among others (**Figure 5**, **Table 4**).

**Table 4:** Mendelian randomization analyses of the causal effects between immune cells, plasma metabolites and COPD.

Immune cell	Metabolite	Outcome	Mediated effect	Mediated proportion	Beta Direct	P-val
CD62L- myeloid DC AC	1-palmitoyl-GPG (16:0) levels	COPD	0.00431	10%	0.039	0.041
HLA DR+ T cell%T cell	Alpha-tocopherol levels	COPD	-0.0029	12.4%	-0.020	0.012
CD28+ CD45RA- CD8br %T cell	Alpha-hydroxyisovalerate levels	COPD	0.00205	14.2%	0.012	0.036
CD24 on memory B cell	1-palmitoyl-GPG (16:0) levels	COPD	0.00267	10%	0.024	0.039
CD24 on memory B cell	1-methylnicotinamide levels	COPD	0.0070	26.4%	0.020	0.013
CD25 on transitional	Cinnamoylglycine levels	COPD	0.00491	14.8%	0.028	0.039
CD27 on unsw mem	Taurochenodeoxycholate levels	COPD	0.00671	18.8%	0.029	0.008
CCR2 on plasmacytoid DC	N-oleoylserine levels	COPD	0.00321	9.09%	0.032	0.040
HLA DR on plasmacytoid DC	X-19438 levels	COPD	0.00424	21.8%	0.015	0.004

## 4 Discussion

In our MR study, the findings indicated a causal relationship between 41 immune cell phenotypes and COPD. However, a reverse MR analysis revealed that 35 immune cell phenotypes bore no causal relationship with COPD, while 6 did exhibit a causal connection. Further employing TSMR and MVMR for mediation analysis, we identified that 8 cell phenotypes could be causally linked to COPD through 8 plasma metabolites (including one unidentified), among which the mediation proportion of 1-methylnicotinamide levels was the highest at 26.4%.

Our research has corroborated the existence of a causal relationship between 41 immune cell phenotypes and COPD, aligning with previous studies that posit chronic inflammation leading to compromised immunity and immunosuppression as pivotal in the pathogenesis of COPD (40), a condition persistently present in the disease (41). It has been observed that, compared to healthy individuals, patients with COPD exhibit an increase in B cells and their products in the blood and lungs (42), alongside an upsurge in the expression of genes related to inflammation, B cell activation, and proliferation. This activation of B cells is associated with an autoimmune-mediated mechanism of COPD pathogenesis (43, 44). However, the association between B cells and COPD does not imply causality (45). Our study, however, confirms a causal relationship between specific B cell phenotypes and COPD, with an increase in memory B-cells being linked to impaired lung function and small airway dysfunction (46), consistent with our findings that CD24 on memory B cells increases the risk of COPD. Furthermore, the subgroups of peripheral blood TBNK lymphocytes in COPD patients show a certain correlation with COPD (47) and its severity (48), aligning with our MR results and suggesting a causal relationship. Regulatory T (Treg) cells play a crucial role in the immune system by suppressing excessive immune responses and maintaining immune balance. The relationship between Treg cells and lung function (49), the imbalance of Treg cells during COPD progression (50), and the potential of modulating Treg cells to improve COPD (51) and lung inflammation underscore their significance (52). Myeloid cells, capable of phagocytosing pathogens, initiating inflammatory responses, and presenting antigens to other immune cells, contribute to tissue repair and remodeling. Our analysis identified six immune cell phenotypes with a causal relationship to COPD in both directions, namely CD14+ CD16+ monocyte AC, CD4+ CD8dim %lymphocyte, CD4+ CD8dim %leukocyte, CD3- lymphocyte AC, CD3 on EM CD8br, and CD45 on Im MDS, all associated with a reduced risk of COPD. The CD14+ CD16+ monocyte AC, a monocyte subgroup expressing CD14 and CD16, plays a role in modulating inflammatory responses and promoting tissue repair, with monocytes being etiologically related to COPD (53) and influencing its pathogenesis and diagnosis (54, 55), serving as key drivers of lung inflammation and tissue remodeling (56). The CD4+ CD8dim %lymphocyte, a unique lymphocyte, plays a role in regulating immune responses and maintaining immune balance, with CD4-regulated T cells controlling autoimmunity and thus managing lung inflammation in COPD (57, 58), while CD4 and CD8 are related to bronchiolar wall remodeling in COPD (59) and the reduction of terminal bronchioles (60).

Metabolites play a crucial role in the early identification of individuals at high risk and in the prevention of diseases (61). Clinically, they enable us to differentiate the disease characteristics of COPD (62), identify diagnostic biomarkers (63, 64), and evaluate the efficacy indicators of COPD treatments (65). Our study has discovered a causal relationship between CD24 on memory B cells and COPD, mediated by two intermediaries: 1-methylnicotinamide and 1-palmitoyl-GPG (16:0). There is a positive correlation between CD24 on memory B cells and COPD, where an increase in CD24 on memory B cells elevates the risk of COPD. Furthermore, CD24 on memory B cells is positively correlated with 1-methylnicotinamide, which, in turn, is positively associated with COPD. 1-methylnicotinamide, a primary metabolite found in all living organisms and involved in growth, development, or reproduction, possesses various immunomodulatory properties. It is linked to inflammatory responses in lung epithelial cells (66)and the activation of the NLRP3 inflammasome (67), a significant mediator in COPD inflammation (68). Through NLRP3, lung inflammation can be regulated (69, 70), indicating a certain correlation between 1-methylnicotinamide and COPD. Our research confirms a causal relationship between 1-methylnicotinamide and COPD, with CD24 on memory B cells influencing COPD risk through the mediation of 1-methylnicotinamide. Conversely, CD24 on memory B cells is negatively correlated with 1-palmitoyl-GPG (16:0), which is positively associated with COPD. Research on 1-palmitoyl-GPG (16:0) is limited, but it is generally considered part of lipid metabolism, related to vitamin D deficiency (71) and pulmonary hypertension (72). Vitamin D may be a risk factor for COPD (73), though specific studies on COPD are lacking. Lipid metabolism plays a key role in the adaptive immune response to chronic inflammation (74) and is associated with lung inflammation in mice (75), with COPD patients exhibiting higher lipid expression (76). Our MR analysis concludes a causal relationship between CD24 on memory B cells and COPD through 1-palmitoyl-GPG (16:0).

Taurochenodeoxycholate could potentially serve as an intermediary in the causal relationship between CD27 on unsw mem and COPD, demonstrating a negative correlation with both CD27 on unsw mem and COPD. Taurochenodeoxycholate, a bile acid functionally related to chenodeoxycholic acid, is involved in inflammatory responses (77), immune cell regulation (78), endoplasmic reticulum stress inhibition (79), and is associated with pulmonary fibrosis (80). However, research specifically targeting its role in COPD is scarce. MR analysis suggests that CD27 on unsw mem may have a causal relationship with COPD through the mediation of taurochenodeoxycholate. Alpha-tocopherol, the most active form of Vitamin E, has been observed in studies to reduce the risk of COPD in women (81), exhibiting anti-inflammatory and antioxidant properties that improve bronchial epithelial thickening, alveolar destruction, and lung function (82). Consistent with our MR analysis, alpha-tocopherol is negatively correlated with the risk of COPD, mediating a causal relationship between COPD and the percentage of HLA DR+ T cells among T cells. Alpha-hydroxyisovalerate, initially identified in studies related to human aging and early development (83), is associated with the severity of bronchiolitis (84) and, consistent with our MR analysis, positively correlated with the risk of COPD. It mediates a causal relationship between COPD and the phenotype of CD28+ CD45RA- CD8 bright %T cells. Cinnamoylglycine, with limited research related to COPD, has been found through MR analysis to be positively correlated with COPD. CD25 on transitional cells has a causal relationship with COPD mediated by cinnamoylglycine. N-oleoylserine, a secondary metabolite functionally related to oleic acid and with scant research in the context of lung inflammation (85), has been found through MR analysis to be negatively correlated with COPD, suggesting a protective factor.

## 5 Conclusion

This study represents a comprehensive assessment of the causal relationships between immune cell phenotypes, plasma metabolites, and COPD. We have identified 8 immune cell phenotypes that exhibit a causal relationship with COPD, mediated by 8 metabolites. These findings illuminate the significance of the underlying mechanisms between immune cells, metabolites, and COPD. They contribute to the screening of individuals at high risk for COPD and offer insights into early prevention and the preemptive diagnosis of Pre-COPD conditions.

## Data availability statement

The original contributions presented in the study are included in the article/supplementary material. Further inquiries can be directed to the corresponding author.

## Ethics statement

Ethical approval was not required for the study involving humans in accordance with the local legislation and institutional requirements. Written informed consent to participate in this study was not required from the participants or the participants’ legal guardians/next of kin in accordance with the national legislation and the institutional requirements.

## Author contributions

ZC: Writing – review & editing, Writing – original draft, Conceptualization. TW: Writing – original draft, Data curation. YF: Writing – original draft, Data curation. FS: Writing – original draft, Supervision, Data curation. HD: Writing – original draft, Data curation. LZ: Writing – original draft, Data curation. LS: Writing – review & editing, Writing – original draft, Conceptualization.

## References

[1] Global Initiative for Chronic Obstructive Lung Disease (Gold). Global Strategy for the Diagnosis, Management and Prevention of Chronic Obstructive Lung Disease (2024 Report) . Available online at: https://Goldcopd.Org/

[2] Collaborators GBDCRD. Prevalence and attributable health burden of chronic respiratory diseases, 1990-2017: A systematic analysis for the global burden of disease study 2017. Lancet Respir Med. (2020) 8:585–96. doi: 10.1016/S2213-2600(20)30105-3 PMC7284317 32526187

[3] Who. Global Health Estimates: Leading Causes of Death. Cause-Specific Mortality 2000–2019 . Available online at: https://Www.Who.Int/Data/Gho/Data/Themes/Mortality-and-Global-Health-Estimates/Ghe-Leading-Causes-of-Death

[4] Adeloye D Song P Zhu Y Campbell H Sheikh A Rudan I. Global, Regional, and National Prevalence of, and Risk Factors for, Chronic Obstructive Pulmonary Disease (COPD) in 2019: A Systematic Review and Modelling Analysis. Lancet Respir Med. (2022) 10:447–58. doi: 10.1016/S2213-2600(21)00511-7 PMC9050565 35279265

[5] Meghji J Mortimer K Agusti A Allwood BW Asher I Bateman ED. Improving lung health in low-income and middle-income countries: from challenges to solutions. Lancet. (2021) 397:928–40. doi: 10.1016/S0140-6736(21)00458-X 33631128

[6] Aegerter H Lambrecht BN Jakubzick CV. Biology of lung macrophages in health and disease. Immunity. (2022) 55:1564–80. doi: 10.1016/j.immuni.2022.08.010 PMC9533769 36103853

[7] Planer JD Morrisey EE. After the storm: regeneration, repair, and reestablishment of homeostasis between the alveolar epithelium and innate immune system following viral lung injury. Annu Rev Pathol. (2023) 18:337–59. doi: 10.1146/annurev-pathmechdis-031621-024344 PMC10875627 36270292

[8] Bracke KR Polverino F. Blunted adaptive immune responses and acute exacerbations of COPD: breaking the code. Eur Respir J. (2023) 62. doi: 10.1183/13993003.01030-2023 37536726

[9] Kapellos TS Conlon TM Yildirim AO Lehmann M. The impact of the immune system on lung injury and regeneration in Copd. Eur Respir J. (2023) 62. doi: 10.1183/13993003.00589-2023 37652569

[10] Barnes PJ. Inflammatory mechanisms in patients with chronic obstructive pulmonary disease. J Allergy Clin Immunol. (2016) 138:16–27. doi: 10.1016/j.jaci.2016.05.011 27373322

[11] Kalathil SG Lugade AA Pradhan V Miller A Parameswaran GI Sethi S. T-regulatory cells and programmed death 1+ T cells contribute to effector T-cell dysfunction in patients with chronic obstructive pulmonary disease. Am J Respir Crit Care Med. (2014) 190:40–50. doi: 10.1164/rccm.201312-2293OC 24825462 PMC4226027

[12] Polverino F Cosio BG Pons J Laucho-Contreras M Tejera P Iglesias A. B cell-activating factor. An orchestrator of lymphoid follicles in severe chronic obstructive pulmonary disease. Am J Respir Crit Care Med. (2015) 192:695–705. doi: 10.1164/rccm.201501-0107OC 26073875 PMC4595676

[13] Villasenor-Altamirano AB Jain D Jeong Y Menon JA Kamiya M Haider H. Activation of Cd8(+) T cells in chronic obstructive pulmonary disease lung. Am J Respir Crit Care Med. (2023) 208:1177–95. doi: 10.1164/rccm.202305-0924OC PMC10868372 37756440

[14] de Fays C Geudens V Gyselinck I Kerckhof P Vermaut A Goos T. Mucosal immune alterations at the early onset of tissue destruction in chronic obstructive pulmonary disease. Front Immunol. (2023) 14:1275845. doi: 10.3389/fimmu.2023.1275845 37915582 PMC10616299

[15] Polverino F Kalhan R. Leveraging omics to predict chronic obstructive pulmonary disease exacerbations: the "Immunome". Am J Respir Crit Care Med. (2023) 208:220–2. doi: 10.1164/rccm.202306-0978ED PMC10395726 37352489

[16] Ryu MH Yun JH Morrow JD Saferali A Castaldi P Chase R. Blood gene expression and immune cell subtypes associated with chronic obstructive pulmonary disease exacerbations. Am J Respir Crit Care Med. (2023) 208:247–55. doi: 10.1164/rccm.202301-0085OC PMC10395718 37286295

[17] Wang L Tang Y Liu S Mao S Ling Y Liu D. Metabonomic profiling of serum and urine by (1)H nmr-based spectroscopy discriminates patients with chronic obstructive pulmonary disease and healthy individuals. PLoS One. (2013) 8:e65675. doi: 10.1371/journal.pone.0065675 23755267 PMC3675021

[18] Chen Y Lu T Pettersson-Kymmer U Stewart ID Butler-Laporte G Nakanishi T. Genomic atlas of the plasma metabolome prioritizes metabolites implicated in human diseases. Nat Genet. (2023) 55:44–53. doi: 10.1038/s41588-022-01270-1 36635386 PMC7614162

[19] Ibrahim W Wilde MJ Cordell RL Richardson M Salman D Free RC. Visualization of exhaled breath metabolites reveals distinct diagnostic signatures for acute cardiorespiratory breathlessness. Sci Transl Med. (2022) 14:eabl5849. doi: 10.1126/scitranslmed.abl5849 36383685 PMC7613858

[20] Madapoosi SS Cruickshank-Quinn C Opron K Erb-Downward JR Begley LA Li G. Lung microbiota and metabolites collectively associate with clinical outcomes in milder stage chronic obstructive pulmonary disease. Am J Respir Crit Care Med. (2022) 206:427–39. doi: 10.1164/rccm.202110-2241OC PMC11418810 35536732

[21] Antunes KH Singanayagam A Williams L Faiez TS Farias A Jackson MM. Airway-delivered short-chain fatty acid acetate boosts antiviral immunity during rhinovirus infection. J Allergy Clin Immunol. (2023) 151:447–57.e5. doi: 10.1016/j.jaci.2022.09.026 36216081

[22] Zhou J Li Q Liu C Pang R Yin Y. Plasma metabolomics and lipidomics reveal perturbed metabolites in different disease stages of chronic obstructive pulmonary disease. Int J Chron Obstruct Pulmon Dis. (2020) 15:553–65. doi: 10.2147/COPD.S229505 PMC7073598 32210549

[23] Bowler RP Jacobson S Cruickshank C Hughes GJ Siska C Ory DS. Plasma sphingolipids associated with chronic obstructive pulmonary disease phenotypes. Am J Respir Crit Care Med. (2015) 191:275–84. doi: 10.1164/rccm.201410-1771OC PMC4351578 25494452

[24] Emdin CA Khera AV Kathiresan S. Mendelian randomization. JAMA. (2017) 318:1925–6. doi: 10.1001/jama.2017.17219 29164242

[25] Orru V Steri M Sidore C Marongiu M Serra V Olla S. Complex genetic signatures in immune cells underlie autoimmunity and inform therapy. Nat Genet. (2020) 52:1036–45. doi: 10.1038/s41588-020-0684-4 PMC8517961 32929287

[26] Burgess S Scott RA Timpson NJ Davey Smith G Thompson SG Consortium E-I. Using published data in Mendelian randomization: A blueprint for efficient identification of causal risk factors. Eur J Epidemiol. (2015) 30:543–52. doi: 10.1007/s10654-015-0011-z PMC4516908 25773750

[27] He M Xu C Yang R Liu L Zhou D Yan S. Causal relationship between human blood metabolites and risk of ischemic stroke: A Mendelian randomization study. Front Genet. (2024) 15:1333454. doi: 10.3389/fgene.2024.1333454 38313676 PMC10834680

[28] Yang J Yan B Zhao B Fan Y He X Yang L. Assessing the causal effects of human serum metabolites on 5 major psychiatric disorders. Schizophr Bull. (2020) 46:804–13. doi: 10.1093/schbul/sbz138 PMC7342080 31919502

[29] Ran B Qin J Wu Y Wen F. Causal role of immune cells in chronic obstructive pulmonary disease: Mendelian randomization study. Expert Rev Clin Immunol. (2024) 20:413–21. doi: 10.1080/1744666X.2023.2295987 38108202

[30] Yuan J Xiong X Zhang B Feng Q Zhang J Wang W. Genetically predicted C-reactive protein mediates the association between rheumatoid arthritis and Atlantoaxial subluxation. Front Endocrinol (Lausanne). (2022) 13:1054206. doi: 10.3389/fendo.2022.1054206 36589832 PMC9800511

[31] Choi KW Chen CY Stein MB Klimentidis YC Wang MJ Koenen KC. Assessment of bidirectional relationships between physical activity and depression among adults: A 2-sample Mendelian randomization study. JAMA Psychiatry. (2019) 76:399–408. doi: 10.1001/jamapsychiatry.2018.4175 30673066 PMC6450288

[32] Cheng ZX Hua JL Jie ZJ Li XJ Zhang J. Genetic insights into the gut-lung axis: Mendelian randomization analysis on gut microbiota, lung function, and COPD. Int J Chron Obstruct Pulmon Dis. (2024) 19:643–53. doi: 10.2147/COPD.S441242 PMC10921945 38464560

[33] Pierce BL Burgess S. Efficient design for Mendelian randomization studies: subsample and 2-sample instrumental variable estimators. Am J Epidemiol. (2013) 178:1177–84. doi: 10.1093/aje/kwt084 PMC3783091 23863760

[34] Davies NM Holmes MV Davey Smith G. Reading Mendelian randomisation studies: A guide, glossary, and checklist for clinicians. BMJ. (2018) 362:k601. doi: 10.1136/bmj.k601 30002074 PMC6041728

[35] Sanderson E. Multivariable Mendelian randomization and mediation. Cold Spring Harb Perspect Med. (2021) 11. doi: 10.1101/cshperspect.a038984 PMC7849347 32341063

[36] Burgess S Thompson SG. Erratum to: interpreting findings from Mendelian randomization using the Mr-Egger method. Eur J Epidemiol. (2017) 32:391–2. doi: 10.1007/s10654-017-0276-5 PMC5506233 28527048

[37] Cai J Li X Wu S Tian Y Zhang Y Wei Z. Assessing the causal association between human blood metabolites and the risk of epilepsy. J Transl Med. (2022) 20:437. doi: 10.1186/s12967-022-03648-5 36180952 PMC9524049

[38] Shi Y Feng S Yan M Wei S Yang K Feng Y. Inflammatory bowel disease and celiac disease: A bidirectional Mendelian randomization study. Front Genet. (2022) 13:928944. doi: 10.3389/fgene.2022.928944 36061176 PMC9437575

[39] Carter AR Sanderson E Hammerton G Richmond RC Davey Smith G Heron J. Mendelian randomisation for mediation analysis: current methods and challenges for implementation. Eur J Epidemiol. (2021) 36:465–78. doi: 10.1007/s10654-021-00757-1 PMC8159796 33961203

[40] Bhat TA Panzica L Kalathil SG Thanavala Y. Immune dysfunction in patients with chronic obstructive pulmonary disease. Ann Am Thorac Soc. (2015) 12 Suppl 2:S169–75. doi: 10.1513/AnnalsATS.201503-126AW PMC4722840 26595735

[41] Brusselle GG Joos GF Bracke KR. New insights into the immunology of chronic obstructive pulmonary disease. Lancet. (2011) 378:1015–26. doi: 10.1016/S0140-6736(11)60988-4 21907865

[42] Nunez B Sauleda J Anto JM Julia MR Orozco M Monso E. Anti-tissue antibodies are related to lung function in chronic obstructive pulmonary disease. Am J Respir Crit Care Med. (2011) 183:1025–31. doi: 10.1164/rccm.201001-0029OC 21097696

[43] Rojas-Quintero J Ochsner SA New F Divakar P Yang CX Wu TD. Spatial transcriptomics resolve an emphysema-specific lymphoid follicle B cell signature in chronic obstructive pulmonary disease. Am J Respir Crit Care Med. (2024) 209:48–58. doi: 10.1164/rccm.202303-0507LE 37934672 PMC10870877

[44] Faner R Cruz T Casserras T Lopez-Giraldo A Noell G Coca I. Network analysis of lung transcriptomics reveals a distinct B-cell signature in emphysema. Am J Respir Crit Care Med. (2016) 193:1242–53. doi: 10.1164/rccm.201507-1311OC 26735770

[45] Sullivan JL Bagevalu B Glass C Sholl L Kraft M Martinez FD. B cell-adaptive immune profile in emphysema-predominant chronic obstructive pulmonary disease. Am J Respir Crit Care Med. (2019) 200:1434–9. doi: 10.1164/rccm.201903-0632LE PMC6884042 31348682

[46] Habener A Grychtol R Gaedcke S DeLuca D Dittrich AM Happle C. Iga(+) memory B-cells are significantly increased in patients with asthma and small airway dysfunction. Eur Respir J. (2022) 60. doi: 10.1183/13993003.02130-2021 PMC9630610 35595320

[47] Hong X Xiao Z. Changes in peripheral blood Tbnk lymphocyte subsets and their association with acute exacerbation of chronic obstructive pulmonary disease. J Int Med Res. (2023) 51:3000605231182556. doi: 10.1177/03000605231182556 37382080 PMC10328016

[48] Xue W Ma J Li Y Xie C. Role of Cd(4) (+) T and Cd(8) (+) T lymphocytes-mediated cellular immunity in pathogenesis of chronic obstructive pulmonary disease. J Immunol Res. (2022) 2022:1429213. doi: 10.1155/2022/1429213 35785027 PMC9242747

[49] Kim WD Sin DD. Granzyme B may act as an effector molecule to control the inflammatory process in Copd. COPD. (2024) 21:1–11. doi: 10.1080/15412555.2023.2299104 38314671

[50] Lourenco JD Ito JT Martins MA Tiberio I Lopes F. Th17/Treg imbalance in chronic obstructive pulmonary disease: clinical and experimental evidence. Front Immunol. (2021) 12:804919. doi: 10.3389/fimmu.2021.804919 34956243 PMC8695876

[51] Zhang X Li X Ma W Liu F Huang P Wei L. Astragaloside Iv restores Th17/Treg balance *via* inhibiting Cxcr4 to improve chronic obstructive pulmonary disease. Immunopharmacol Immunotoxicol. (2023) 45:682–91. doi: 10.1080/08923973.2023.2228479 37417915

[52] Li Y Shen D Wang K Xue Y Liu J Li S. Mogroside V ameliorates broiler pulmonary inflammation *via* modulating lung microbiota and rectifying Th17/Treg dysregulation in lipopolysaccharides-induced lung injury. Poult Sci. (2023) 102:103138. doi: 10.1016/j.psj.2023.103138 37862871 PMC10590742

[53] Lee Y Song J Jeong Y Choi E Ahn C Jang W. Meta-analysis of single-cell Rna-sequencing data for depicting the transcriptomic landscape of chronic obstructive pulmonary disease. Comput Biol Med. (2023) 167:107685. doi: 10.1016/j.compbiomed.2023.107685 37976829

[54] Huang W Luo T Lan M Zhou W Zhang M Wu L. Identification and characterization of a Cerna regulatory network involving Linc00482 and Prrc2b in peripheral blood mononuclear cells: implications for Copd pathogenesis and diagnosis. Int J Chron Obstruct Pulmon Dis. (2024) 19:419–30. doi: 10.2147/COPD.S437046 PMC10860591 38348310

[55] Hu Y Shao X Xing L Li X Nonis GM Koelwyn GJ. Single-cell sequencing of lung macrophages and monocytes reveals novel therapeutic targets in COPD. Cells. (2023) 12. doi: 10.3390/cells12242771 PMC10741950 38132091

[56] Wohnhaas CT Bassler K Watson CK Shen Y Leparc GG Tilp C. Monocyte-derived alveolar macrophages are key drivers of smoke-induced lung inflammation and tissue remodeling. Front Immunol. (2024) 15:1325090. doi: 10.3389/fimmu.2024.1325090 38348034 PMC10859862

[57] Smyth LJ Starkey C Vestbo J Singh D. Cd4-regulatory cells in Copd patients. Chest. (2007) 132:156–63. doi: 10.1378/chest.07-0083 17505034

[58] Forsslund H Mikko M Karimi R Grunewald J Wheelock AM Wahlstrom J. Distribution of T-cell subsets in Bal fluid of patients with mild to moderate Copd depends on current smoking status and not airway obstruction. Chest. (2014) 145:711–22. doi: 10.1378/chest.13-0873 24264182

[59] Booth S Hsieh A Mostaco-Guidolin L Koo HK Wu K Aminazadeh F. A single-cell atlas of small airway disease in chronic obstructive pulmonary disease: A cross-sectional study. Am J Respir Crit Care Med. (2023) 208:472–86. doi: 10.1164/rccm.202303-0534OC 37406359

[60] Xu F Vasilescu DM Kinose D Tanabe N Ng KW Coxson HO. The molecular and cellular mechanisms associated with the destruction of terminal bronchioles in Copd. Eur Respir J. (2022) 59. doi: 10.1183/13993003.01411-2021 34675046

[61] Buergel T Steinfeldt J Ruyoga G Pietzner M Bizzarri D Vojinovic D. Metabolomic profiles predict individual multidisease outcomes. Nat Med. (2022) 28:2309–20. doi: 10.1038/s41591-022-01980-3 PMC9671812 36138150

[62] Adamko DJ Nair P Mayers I Tsuyuki RT Regush S Rowe BH. Metabolomic profiling of asthma and chronic obstructive pulmonary disease: A pilot study differentiating diseases. J Allergy Clin Immunol. (2015) 136:571–80.e3. doi: 10.1016/j.jaci.2015.05.022 26152317

[63] Li J Liu X Shi Y Xie Y Yang J Du Y. Differentiation in Tcm patterns of chronic obstructive pulmonary disease by comprehensive metabolomic and lipidomic characterization. Front Immunol. (2023) 14:1208480. doi: 10.3389/fimmu.2023.1208480 37492573 PMC10363632

[64] Bowerman KL Rehman SF Vaughan A Lachner N Budden KF Kim RY. Disease-associated gut microbiome and metabolome changes in patients with chronic obstructive pulmonary disease. Nat Commun. (2020) 11:5886. doi: 10.1038/s41467-020-19701-0 33208745 PMC7676259

[65] Hailong Z Yimei S Yan D Xinguang L Jiansheng L. Exploration of biomarkers for efficacy evaluation of traditional Chinese medicine syndromes of acute exacerbation of chronic obstructive pulmonary disease based on metabolomics. Front Pharmacol. (2024) 15:1302950. doi: 10.3389/fphar.2024.1302950 38344179 PMC10853405

[66] Chou PJ Sarwar MS Wang L Wu R Li S Hudlikar RR. Metabolomic, DNA methylomic, and transcriptomic profiling of suberoylanilide hydroxamic acid effects on lps-exposed lung epithelial cells. Cancer Prev Res (Phila). (2023) 16:321–32. doi: 10.1158/1940-6207.CAPR-22-0384 PMC10238674 36867722

[67] Sidor K Jeznach A Hoser G Skirecki T. 1-methylnicotinamide (1-mna) inhibits the activation of the nlrp3 inflammasome in human macrophages. Int Immunopharmacol. (2023) 121:110445. doi: 10.1016/j.intimp.2023.110445 37290319

[68] Pauwels NS Bracke KR Dupont LL Van Pottelberge GR Provoost S Vanden Berghe T. Role of Il-1alpha and the Nlrp3/Caspase-1/Il-1beta axis in cigarette smoke-induced pulmonary inflammation and Copd. Eur Respir J. (2011) 38:1019–28. doi: 10.1183/09031936.00158110 21622588

[69] Li M Hua Q Shao Y Zeng H Liu Y Diao Q. Circular Rna Circbbs9 promotes pm(2.5)-induced lung inflammation in mice. Via Nlrp3 Inflammasome Activation. Environ Int. (2020) 143:105976. doi: 10.1016/j.envint.2020.105976 32707273

[70] Sayan M Mossman BT. The Nlrp3 inflammasome in pathogenic particle and fibre-associated lung inflammation and diseases. Part Fibre Toxicol. (2016) 13:51. doi: 10.1186/s12989-016-0162-4 27650313 PMC5029018

[71] Chatterjee I Lu R Zhang Y Zhang J Dai Y Xia Y. Vitamin D receptor promotes healthy microbial metabolites and microbiome. Sci Rep. (2020) 10:7340. doi: 10.1038/s41598-020-64226-7 32355205 PMC7192915

[72] Heresi GA Mey JT Bartholomew JR Haddadin IS Tonelli AR Dweik RA. Plasma metabolomic profile in chronic thromboembolic pulmonary hypertension. Pulm Circ. (2020) 10:2045894019890553. doi: 10.1177/2045894019890553 32110382 PMC7000865

[73] Hanson C Rutten EP Wouters EF Rennard S. Diet and vitamin D as risk factors for lung impairment and Copd. Transl Res. (2013) 162:219–36. doi: 10.1016/j.trsl.2013.04.004 23685188

[74] Duffney PF Falsetta ML Rackow AR Thatcher TH Phipps RP Sime PJ. Key roles for lipid mediators in the adaptive immune response. J Clin Invest. (2018) 128:2724–31. doi: 10.1172/JCI97951 PMC6025978 30108196

[75] Morissette MC Shen P Thayaparan D Stampfli MR. Disruption of pulmonary lipid homeostasis drives cigarette smoke-induced lung inflammation in mice. Eur Respir J. (2015) 46:1451–60. doi: 10.1183/09031936.00216914 26113683

[76] Telenga ED Hoffmann RF Ruben tK Hoonhorst SJ Willemse BW van Oosterhout AJ. Untargeted lipidomic analysis in chronic obstructive pulmonary disease. Uncovering sphingolipids. Am J Respir Crit Care Med. (2014) 190:155–64. doi: 10.1164/rccm.201312-2210OC 24871890

[77] Nakada EM Bhakta NR Korwin-Mihavics BR Kumar A Chamberlain N Bruno SR. Conjugated bile acids attenuate allergen-induced airway inflammation and hyperresponsiveness by inhibiting Upr transducers. JCI Insight. (2019) 4. doi: 10.1172/jci.insight.98101 PMC6538331 31045581

[78] Li C He YY Zhang YT You YC Yuan HY Wei YG. Tauroursodeoxycholic acid (Tudca) disparate pharmacological effects to lung tissue-resident memory T cells contribute to alleviated silicosis. BioMed Pharmacother. (2022) 151:113173. doi: 10.1016/j.biopha.2022.113173 35623165

[79] Tong B Fu L Hu B Zhang ZC Tan ZX Li SR. Tauroursodeoxycholic acid alleviates pulmonary endoplasmic reticulum stress and epithelial-mesenchymal transition in bleomycin-induced lung fibrosis. BMC Pulm Med. (2021) 21:149. doi: 10.1186/s12890-021-01514-6 33952237 PMC8097922

[80] Tanaka Y Ishitsuka Y Hayasaka M Yamada Y Miyata K Endo M. The exacerbating roles of Ccaat/enhancer-binding protein homologous protein (Chop) in the development of bleomycin-induced pulmonary fibrosis and the preventive effects of tauroursodeoxycholic acid (Tudca) against pulmonary fibrosis in mice. Pharmacol Res. (2015) 99:52–62. doi: 10.1016/j.phrs.2015.05.004 26005208

[81] Agler AH Kurth T Gaziano JM Buring JE Cassano PA. Randomised vitamin E supplementation and risk of chronic lung disease in the women's health study. Thorax. (2011) 66:320–5. doi: 10.1136/thx.2010.155028 PMC3062677 21257986

[82] Peh HY Tan WSD Chan TK Pow CW Foster PS Wong WSF. Vitamin E isoform gamma-tocotrienol protects against emphysema in cigarette smoke-induced Copd. Free Radic Biol Med. (2017) 110:332–44. doi: 10.1016/j.freeradbiomed.2017.06.023 28684161

[83] Menni C Kastenmuller G Petersen AK Bell JT Psatha M Tsai PC. Metabolomic markers reveal novel pathways of ageing and early development in human populations. Int J Epidemiol. (2013) 42:1111–9. doi: 10.1093/ije/dyt094 PMC3781000 23838602

[84] Hasegawa K Stewart CJ Celedon JC Mansbach JM Tierney C Camargo CA Jr. Circulating 25-hydroxyvitamin D, nasopharyngeal airway metabolome, and bronchiolitis severity. Allergy. (2018) 73:1135–40. doi: 10.1111/all.13379 PMC6167253 29315663

[85] Dubuc I Prunier J Lacasse E Gravel A Puhm F Allaeys I. Cytokines and lipid mediators of inflammation in lungs of Sars-Cov-2 infected mice. Front Immunol. (2022) 13:893792. doi: 10.3389/fimmu.2022.893792 35812400 PMC9264370

